# Crystal structure of (*Z*)-2-hy­droxy-*N*′-(4-oxo-1,3-thia­zolidin-2-yl­idene)benzohydrazide

**DOI:** 10.1107/S1600536814022351

**Published:** 2014-10-18

**Authors:** Ya Zhang, Peijuan Li, Xin Fan, Longfei Jin

**Affiliations:** a College of Chemistry and Material Science, South-Central University for Nationalities, Wuhan 430074, People’s Republic of China

**Keywords:** crystal structure, benzohydrazide, 4-thia­zolidinone derivatives, biological activity, hydrogen bonding

## Abstract

In the title compound, C_10_H_9_N_3_O_3_S, the five-membered ring adopts a slightly twisted conformation about the C_m_—S (m = methyl­ene) bond. The dihedral angle between this ring and the benzene ring is 7.99 (9)°. A bifurcated intra­molecular N—H⋯(O,S) hydrogen bond helps to establish the near planar conformation of the mol­ecule. In the crystal, mol­ecules are linked by N—H⋯O and O—H⋯O hydrogen bonds to generate (001) sheets.

## Related literature   

For background to the biological activities of 4-thia­zolidinone derivatives, see: Singh *et al.* (1981 ▶); Verma & Shailendra (2008 ▶); Jain *et al.*, (2012 ▶).
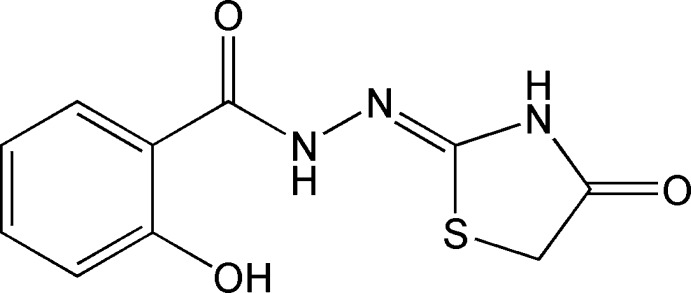


## Experimental   

### Crystal data  

C_10_H_9_N_3_O_3_S
*M* *_r_* = 251.26
Monoclinic, 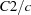

*a* = 18.788 (2) Å
*b* = 8.9334 (10) Å
*c* = 12.7969 (14) Å
β = 92.667 (2)°
*V* = 2145.6 (4) Å^3^
*Z* = 8
Mo *K*α radiation
μ = 0.30 mm^−1^
*T* = 298 K
0.22 × 0.21 × 0.20 mm

### Data collection  

Bruker SMART CCD diffractometer
Absorption correction: multi-scan (*SADABS*; Bruker, 2001 ▶) *T*_min_ = 0.937, *T*_max_ = 0.942
10873 measured reflections
2102 independent reflections
1784 reflections with *I* > 2σ(*I*)
*R*_int_ = 0.032

### Refinement  

*R*[*F* ^2^ > 2σ(*F* ^2^)] = 0.037
*wR*(*F* ^2^) = 0.103
*S* = 0.99
2102 reflections
181 parameters
9 restraints
H atoms treated by a mixture of independent and constrained refinement
Δρ_max_ = 0.36 e Å^−3^
Δρ_min_ = −0.30 e Å^−3^

### 

Data collection: *SMART* (Bruker, 2001 ▶); cell refinement: *SAINT* (Bruker, 2001 ▶); data reduction: *SAINT*; program(s) used to solve structure: *SHELXS97* (Sheldrick, 2008 ▶); program(s) used to refine structure: *SHELXL97* (Sheldrick, 2008 ▶); molecular graphics: *SHELXTL* (Sheldrick, 2008 ▶); software used to prepare material for publication: *SHELXTL*.

## Supplementary Material

Crystal structure: contains datablock(s) global, I. DOI: 10.1107/S1600536814022351/hb7294sup1.cif

Structure factors: contains datablock(s) I. DOI: 10.1107/S1600536814022351/hb7294Isup2.hkl

Supporting information file. DOI: 10.1107/S1600536814022351/hb7294Isup3.cml

**Fig. 1.**
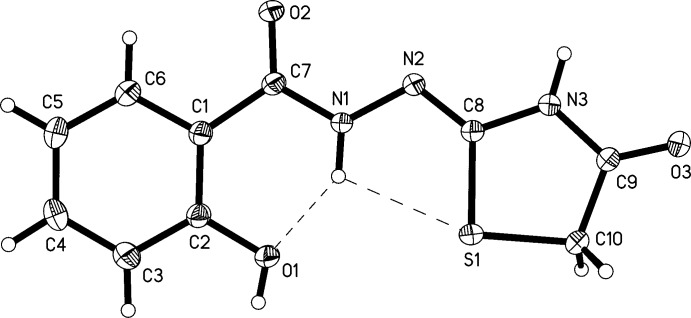
The molecular structure of (I), showing 30% probability displacement ellipsoids. Dashed lines indicate hydrogen bonds.

. DOI: 10.1107/S1600536814022351/hb7294fig1.tif
The mol­ecular structure of (I), showing 30% probability displacement ellipsoids. Dashed lines indicate hydrogen bonds.

**Fig. 2.**
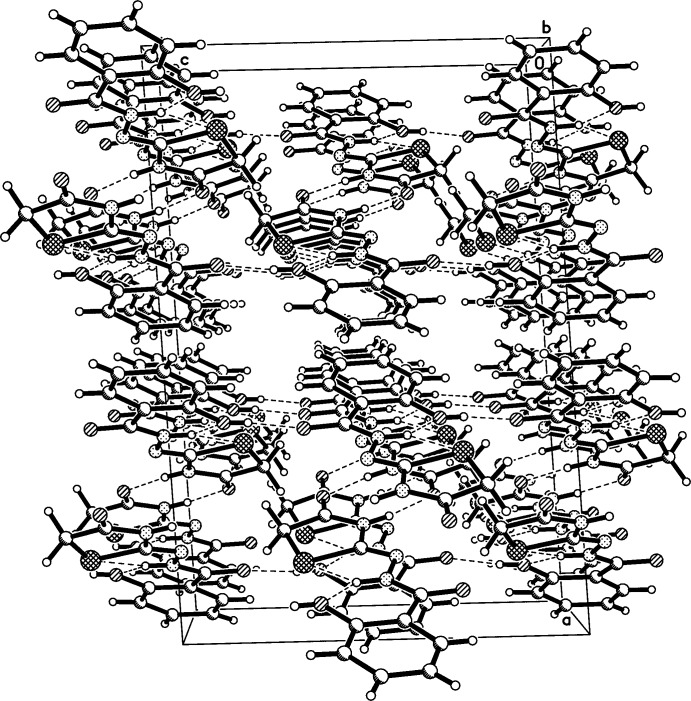
Packing diagram for (I). The hydrogen bonds are indicated by dashed lines.

. DOI: 10.1107/S1600536814022351/hb7294fig2.tif
Packing diagram for (I). The hydrogen bonds are indicated by dashed lines.

CCDC reference: 1028612

Additional supporting information:  crystallographic information; 3D view; checkCIF report

## Figures and Tables

**Table 1:** Hydrogen-bond geometry (, )

*D*H*A*	*D*H	H*A*	*D* *A*	*D*H*A*
N1H1*A*O1	0.91(1)	1.86(1)	2.6166(17)	139(2)
N1H1*A*S1	0.91(1)	2.42(2)	2.8879(15)	112(1)
N3H3*A*O3^i^	0.91(1)	1.88(1)	2.7767(18)	168(2)
O1H1O2^ii^	0.82(1)	1.83(1)	2.6538(16)	177(2)

Symmetry codes: (i) 
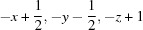
; (ii) 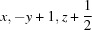.

## supplementary crystallographic information

### S1. Comment

Derivatives of 4-thiazolidinone exhibit prominent biological activites such as antibacterial activity, antifungal activity, antitubercular activity, anticancer activity, antiinflammtory activity, analgesic activity, anticonvulsant activity, antidepressant activity, antiviral/anti-HIV activity, antidiabetic activity, muscarinic receptor 1 agonist, FSH receptor agonist, trypanocidal (anti-epimastigote) activity and antiarrhythmic activity (Jain, *et al.*, 2012; Verma & Shailendra, 2008; Singh *et al.*, 1981). Besides, enough coordinational sites exist in these compounds, lead to a potential to form a supermolecular structure. As part of our ongoing studies, the preparation and X-ray structure determination of the title compound, (I), was undertaken.

In the title molecule (Fig. 1), bond lengths and angles in (I) show normal values. The non-hydrogen atoms of the molecule lie in a plane with an r.m.s deviation of 0.002 Å. An intramolecular tricentered hydrogen bond is observed between the N—H of imino group, O atom of phenolic hydroxyl group and S atom of 4-thiazolidinone group (Fig. 1 and Table 1). The molecules translated one unit cell along the *b* direction are stacked with N3···O3^i^, O1···O2^ii^ and and O3···S1^iii^ [symmetry code: (i) 1/2 - *x*,3/2 - *y*,1 - *z*; (ii) *x*,-*y*,-1/2 + *z*; (iii) 1/2 - *x*,1/2 + *y*,1/2 - *z*] distances of 2.7766 (18) Å, 2.6538 (17) Å and 3.1028 (13) Å, respectively, indicating weak C—H···π interactions.

### S2. Experimental

4-Salicyloyl thiosemicarbazide (2.11 g, 0.01 mol), ethyl bromoacetate (1.67 g, 0.01 mol), sodium acetate (3.28 g, 0.04 mol) and 40 ml of ethyl alcohol were added to a round-bottom flask. Stirred for 10 minutes, then the reaction mixture was slowly warmed to boiling and stirred for 10 h. After cooling to room temperature, 40 ml of water were added and staying for 12 h. The resulting precipitate was filtered and recrystallized with ethyl alcohol to give 1.30 g of the title compound. Colourless blocks were grown by slow evaporation from a mixed solution of methanol+*N*,*N*-dimethyl formamide (6:1) at room temperature.

### S3. Refinement

All H atoms were placed in calculated positions, with N—H distances of 0.91 Å, O—H distances of 0.82 Å, and C—H distances of 0.97 Å (CH_2_) and 0.93 Å (benzyl CH). They were included in the refinement in the riding-model approximation, with isotropic displacement parameters set to 1.2*U*_eq_ of the carrier atom (1.5*U*_eq_ for hydroxyl H atoms).

### Crystal data

**Table:**

C_10_H_9_N_3_O_3_S	*F*(000) = 1040
*M**_r_* = 251.26	*D*_x_ = 1.556 Mg m^−^^3^
Monoclinic, *C*2/*c*	Mo *K*α radiation, λ = 0.71073 Å
Hall symbol: -C 2yc	Cell parameters from 4520 reflections
*a* = 18.788 (2) Å	θ = 2.5–28.0°
*b* = 8.9334 (10) Å	µ = 0.30 mm^−^^1^
*c* = 12.7969 (14) Å	*T* = 298 K
β = 92.667 (2)°	Block, colorless
*V* = 2145.6 (4) Å^3^	0.22 × 0.21 × 0.20 mm
*Z* = 8	

### Data collection

**Table:**

Bruker SMART CCD diffractometer	2102 independent reflections
Radiation source: fine-focus sealed tube	1784 reflections with *I* > 2σ(*I*)
Graphite monochromator	*R*_int_ = 0.032
phi and ω scans	θ_max_ = 26.0°, θ_min_ = 2.2°
Absorption correction: multi-scan (*SADABS*; Bruker, 2001)	*h* = −22→23
*T*_min_ = 0.937, *T*_max_ = 0.942	*k* = −11→11
10873 measured reflections	*l* = −15→15

### Refinement

**Table:**

Refinement on *F*^2^	Primary atom site location: structure-invariant direct methods
Least-squares matrix: full	Secondary atom site location: difference Fourier map
*R*[*F*^2^ > 2σ(*F*^2^)] = 0.037	Hydrogen site location: inferred from neighbouring sites
*wR*(*F*^2^) = 0.103	H atoms treated by a mixture of independent and constrained refinement
*S* = 0.99	*w* = 1/[σ^2^(*F*_o_^2^) + (0.0666*P*)^2^ + 0.9019*P*] where *P* = (*F*_o_^2^ + 2*F*_c_^2^)/3
2102 reflections	(Δ/σ)_max_ = 0.001
181 parameters	Δρ_max_ = 0.36 e Å^−^^3^
9 restraints	Δρ_min_ = −0.30 e Å^−^^3^

### Special details

**Table:**

Geometry. All e.s.d.'s (except the e.s.d. in the dihedral angle between two l.s. planes) are estimated using the full covariance matrix. The cell e.s.d.'s are taken into account individually in the estimation of e.s.d.'s in distances, angles and torsion angles; correlations between e.s.d.'s in cell parameters are only used when they are defined by crystal symmetry. An approximate (isotropic) treatment of cell e.s.d.'s is used for estimating e.s.d.'s involving l.s. planes.
Refinement. Refinement of *F*^2^ against ALL reflections. The weighted *R*-factor *wR* and goodness of fit *S* are based on *F*^2^, conventional *R*-factors *R* are based on *F*, with *F* set to zero for negative *F*^2^. The threshold expression of *F*^2^ > σ(*F*^2^) is used only for calculating *R*-factors(gt) *etc*. and is not relevant to the choice of reflections for refinement. *R*-factors based on *F*^2^ are statistically about twice as large as those based on *F*, and *R*- factors based on ALL data will be even larger.

### Fractional atomic coordinates and isotropic or equivalent isotropic displacement parameters (Å^2^)

**Table:**

	*x*	*y*	*z*	*U*_iso_*/*U*_eq_	
C1	0.40566 (8)	0.54405 (17)	0.47258 (12)	0.0304 (4)	
C2	0.40845 (9)	0.58782 (17)	0.57775 (12)	0.0318 (4)	
C3	0.43987 (10)	0.7222 (2)	0.60795 (14)	0.0422 (4)	
C4	0.46818 (12)	0.8145 (2)	0.53512 (17)	0.0523 (5)	
C5	0.46518 (12)	0.7748 (2)	0.43080 (16)	0.0538 (5)	
C6	0.43446 (11)	0.6417 (2)	0.40054 (14)	0.0421 (4)	
C7	0.37628 (8)	0.39894 (17)	0.43052 (12)	0.0307 (4)	
C8	0.31830 (9)	0.07860 (17)	0.55293 (12)	0.0302 (4)	
C9	0.27480 (9)	−0.13806 (17)	0.63012 (12)	0.0329 (4)	
C10	0.29374 (11)	−0.0495 (2)	0.72704 (13)	0.0398 (4)	
H1	0.3791 (12)	0.539 (2)	0.7078 (9)	0.060*	
H3	0.4396 (10)	0.749 (2)	0.6782 (5)	0.048*	
H4	0.4891 (9)	0.9032 (12)	0.5589 (15)	0.048*	
H5	0.4837 (10)	0.8367 (17)	0.3803 (11)	0.048*	
H6	0.4346 (10)	0.612 (2)	0.3310 (5)	0.048*	
H1A	0.3575 (10)	0.329 (2)	0.5703 (5)	0.048*	
H3A	0.2773 (10)	−0.1079 (19)	0.4796 (7)	0.048*	
H10A	0.3270 (8)	−0.1054 (19)	0.7719 (12)	0.048*	
H10B	0.2520 (6)	−0.022 (2)	0.7644 (14)	0.048*	
N1	0.35652 (8)	0.30049 (15)	0.50199 (10)	0.0338 (3)	
N2	0.32887 (8)	0.16107 (15)	0.47420 (10)	0.0345 (3)	
N3	0.28759 (8)	−0.06155 (15)	0.54179 (10)	0.0346 (3)	
O1	0.38054 (7)	0.49708 (13)	0.65105 (9)	0.0441 (3)	
O2	0.37177 (7)	0.37081 (13)	0.33575 (9)	0.0438 (3)	
O3	0.25005 (7)	−0.26414 (14)	0.63173 (9)	0.0451 (3)	
S1	0.33730 (3)	0.11903 (5)	0.68522 (3)	0.0514 (2)	

### Atomic displacement parameters (Å^2^)

**Table:**

	*U*^11^	*U*^22^	*U*^33^	*U*^12^	*U*^13^	*U*^23^
C1	0.0361 (8)	0.0267 (8)	0.0285 (8)	0.0000 (6)	0.0016 (6)	0.0019 (6)
C2	0.0403 (9)	0.0265 (8)	0.0287 (8)	0.0018 (6)	0.0034 (6)	0.0015 (6)
C3	0.0562 (11)	0.0344 (9)	0.0359 (9)	−0.0042 (8)	0.0016 (8)	−0.0053 (7)
C4	0.0696 (13)	0.0344 (10)	0.0531 (12)	−0.0202 (9)	0.0038 (10)	−0.0048 (9)
C5	0.0753 (14)	0.0414 (11)	0.0455 (12)	−0.0211 (10)	0.0100 (10)	0.0071 (9)
C6	0.0580 (11)	0.0381 (10)	0.0305 (9)	−0.0094 (8)	0.0044 (8)	0.0037 (7)
C7	0.0389 (9)	0.0290 (8)	0.0243 (8)	0.0002 (6)	0.0026 (6)	0.0024 (6)
C8	0.0409 (9)	0.0263 (8)	0.0237 (8)	−0.0013 (6)	0.0029 (6)	−0.0009 (6)
C9	0.0439 (9)	0.0282 (8)	0.0269 (8)	−0.0005 (7)	0.0039 (7)	0.0023 (6)
C10	0.0618 (11)	0.0324 (9)	0.0255 (8)	−0.0068 (8)	0.0053 (7)	0.0032 (7)
N1	0.0555 (9)	0.0249 (7)	0.0211 (7)	−0.0082 (6)	0.0038 (6)	−0.0015 (5)
N2	0.0534 (8)	0.0261 (7)	0.0239 (7)	−0.0070 (6)	0.0012 (6)	0.0002 (5)
N3	0.0540 (9)	0.0267 (7)	0.0230 (7)	−0.0076 (6)	0.0014 (6)	−0.0008 (5)
O1	0.0770 (9)	0.0316 (6)	0.0247 (6)	−0.0106 (6)	0.0122 (6)	−0.0041 (5)
O2	0.0752 (9)	0.0354 (7)	0.0207 (6)	−0.0124 (6)	0.0025 (5)	0.0010 (5)
O3	0.0724 (9)	0.0313 (7)	0.0320 (7)	−0.0149 (6)	0.0060 (6)	0.0027 (5)
S1	0.0938 (4)	0.0391 (3)	0.0215 (2)	−0.0258 (2)	0.0027 (2)	−0.00162 (17)

### Geometric parameters (Å, º)

**Table:**

C1—C6	1.396 (2)	C8—N2	1.271 (2)
C1—C2	1.400 (2)	C8—N3	1.383 (2)
C1—C7	1.499 (2)	C8—S1	1.7514 (16)
C2—O1	1.3630 (19)	C9—O3	1.219 (2)
C2—C3	1.385 (2)	C9—N3	1.352 (2)
C3—C4	1.371 (3)	C9—C10	1.500 (2)
C3—H3	0.930 (2)	C10—S1	1.8064 (18)
C4—C5	1.380 (3)	C10—H10A	0.968 (4)
C4—H4	0.930 (2)	C10—H10B	0.969 (4)
C5—C6	1.370 (3)	N1—N2	1.3893 (18)
C5—H5	0.930 (2)	N1—H1A	0.909 (2)
C6—H6	0.930 (2)	N3—H3A	0.909 (2)
C7—O2	1.2376 (19)	O1—H1	0.820 (2)
C7—N1	1.334 (2)	O1—H1	0.820 (2)
			
C6—C1—C2	117.51 (15)	N2—C8—S1	127.90 (13)
C6—C1—C7	116.79 (14)	N3—C8—S1	110.58 (11)
C2—C1—C7	125.68 (14)	O3—C9—N3	124.33 (15)
O1—C2—C3	119.75 (15)	O3—C9—C10	123.33 (15)
O1—C2—C1	119.81 (14)	N3—C9—C10	112.33 (14)
C3—C2—C1	120.45 (15)	C9—C10—S1	106.74 (11)
C4—C3—C2	120.40 (17)	C9—C10—H10A	109.9 (12)
C4—C3—H3	121.5 (13)	S1—C10—H10A	108.5 (12)
C2—C3—H3	118.1 (13)	C9—C10—H10B	112.0 (12)
C3—C4—C5	120.22 (17)	S1—C10—H10B	109.0 (12)
C3—C4—H4	117.6 (13)	H10A—C10—H10B	110.6 (17)
C5—C4—H4	122.2 (13)	C7—N1—N2	121.89 (13)
C6—C5—C4	119.64 (17)	C7—N1—H1A	118.8 (13)
C6—C5—H5	119.1 (13)	N2—N1—H1A	119.1 (13)
C4—C5—H5	121.3 (13)	C8—N2—N1	112.77 (13)
C5—C6—C1	121.79 (17)	C9—N3—C8	117.45 (13)
C5—C6—H6	120.1 (12)	C9—N3—H3A	117.5 (12)
C1—C6—H6	118.0 (12)	C8—N3—H3A	125.0 (12)
O2—C7—N1	121.96 (15)	H1—O1—C2	111.5 (16)
O2—C7—C1	122.33 (14)	C2—O1—H1	111.5 (16)
N1—C7—C1	115.70 (13)	C8—S1—C10	92.28 (8)
N2—C8—N3	121.51 (14)		
			
C6—C1—C2—O1	179.46 (16)	N3—C9—C10—S1	−7.08 (19)
C7—C1—C2—O1	−2.4 (3)	O2—C7—N1—N2	−0.8 (3)
C6—C1—C2—C3	−1.1 (3)	C1—C7—N1—N2	−179.53 (14)
C7—C1—C2—C3	177.05 (16)	N3—C8—N2—N1	176.65 (14)
O1—C2—C3—C4	179.98 (18)	S1—C8—N2—N1	−2.5 (2)
C1—C2—C3—C4	0.5 (3)	C7—N1—N2—C8	174.89 (15)
C2—C3—C4—C5	0.4 (3)	O3—C9—N3—C8	−177.67 (16)
C3—C4—C5—C6	−0.7 (4)	C10—C9—N3—C8	3.0 (2)
C4—C5—C6—C1	0.1 (3)	N2—C8—N3—C9	−176.46 (16)
C2—C1—C6—C5	0.8 (3)	S1—C8—N3—C9	2.81 (19)
C7—C1—C6—C5	−177.51 (18)	C3—C2—O1—H1	9.9 (17)
C6—C1—C7—O2	−6.1 (2)	C1—C2—O1—H1	−170.6 (17)
C2—C1—C7—O2	175.83 (16)	N2—C8—S1—C10	173.24 (17)
C6—C1—C7—N1	172.68 (15)	N3—C8—S1—C10	−5.97 (13)
C2—C1—C7—N1	−5.4 (2)	C9—C10—S1—C8	7.23 (14)
O3—C9—C10—S1	173.59 (15)		

### Hydrogen-bond geometry (Å, º)

**Table:**

*D*—H···*A*	*D*—H	H···*A*	*D*···*A*	*D*—H···*A*
N1—H1*A*···O1	0.91 (1)	1.86 (1)	2.6166 (17)	139 (2)
N1—H1*A*···S1	0.91 (1)	2.42 (2)	2.8879 (15)	112 (1)
N3—H3*A*···O3^i^	0.91 (1)	1.88 (1)	2.7767 (18)	168 (2)
O1—H1···O2^ii^	0.82 (1)	1.83 (1)	2.6538 (16)	177 (2)

Symmetry codes: (i) −*x*+1/2, −*y*−1/2, −*z*+1; (ii) *x*, −*y*+1, *z*+1/2.
